# Seasonal variation in non-structural carbohydrates, sucrolytic activity and secondary metabolites in deciduous and perennial *Diospyros* species sampled in Western Mexico

**DOI:** 10.1371/journal.pone.0187235

**Published:** 2017-10-26

**Authors:** Ernesto Ramírez-Briones, Ramón Rodríguez-Macías, Eduardo Salcedo-Pérez, Norma Martínez-Gallardo, Axel Tiessen, Jorge Molina-Torres, John P. Délano-Frier, Julia Zañudo-Hernández

**Affiliations:** 1 Centro Universitario de Ciencias Biológicas y Agropecuarias, Camino Ing., La Venta del Astillero, Zapopan, Jalisco, México; 2 Centro Universitario de Ciencias Exactas e Ingeniería, Camino Ing., La Venta del Astillero, Zapopan, Jalisco, México; 3 Centro de Investigación y de Estudios Avanzados del Instituto Politécnico Nacional, Unidad Irapuato, Libramiento Norte Carretera Irapuato León Kilómetro 9.6, Carretera Irapuato León, Irapuato, Guanajuato, México; Austrian Federal Research Centre for Forests BFW, AUSTRIA

## Abstract

This study was performed to test the working hypothesis that the primary determinants influencing seasonal driven modifications in carbon mobilization and other key biochemical parameters in leaves of poorly known *Diospyros digyna* (Ddg; semi-domesticated; perennial) and *D*. *rekoi* (Dre; undomesticated; deciduous) trees are determined by environmental growing conditions, agronomic management and physiological plasticity. Thus, biochemical changes in leaves of both trees were recorded seasonally during two successive fruiting years. Trees were randomly sampled in Western Mexico habitats with differing soil quality, climatic conditions, luminosity, and cultivation practices. Leaves of Ddg had consistently higher total chlorophyll contents (C_T_) that, unexpectedly, peaked in the winter of 2015. In Dre, the highest leaf C_T_ values recorded in the summer of 2015 inversely correlated with low average luminosity and high Chl a/ Chlb ratios. The seasonal C_T_ variations in Dre were congruent with varying luminosity, whereas those in Ddg were probably affected by other factors, such as fluctuating leaf protein contents and the funneling of light energy to foliar non-structural carbohydrates (NSCs) accumulation, which were consistently higher than those detected in Dre leaves. Seasonal foliar NSC fluctuations in both species were in agreement with the carbon (C) demands of flowering, fruiting and/ or leaf regrowth. Seasonal changes in foliar hexose to sucrose (Hex/ Suc) ratios coincided with cell wall invertase activity in both species. In Dre, high Hex/ Suc ratios in spring leaves possibly allowed an accumulation of phenolic acids, not observed in Ddg. The above results supported the hypothesis proposed by showing that leaf responses to changing environmental conditions differ in perennial and deciduous *Diospyros* trees, including a dynamic adjustment of NSCs to supply the C demands imposed by reproduction, leaf regrowth and, possibly, stress.

## Introduction

Plants are constantly challenged by variations in temperature, rainfall, and luminosity during their life cycle. Thus, in order to maintain optimal rates of development under fluctuating ambient conditions, they must employ mechanisms designed to maintain photosynthetic efficiency, as well as to ensure the proper functioning of vital metabolic cycles together with membrane and organelle integrity [1, 2]. In this context, plant carbohydrates constitute central players in plant metabolism, not only as primordial sources of energy, but also as signal conveying molecules. They can be classified as structural or non-structural carbohydrates (NSCs), the latter of which include water-soluble glucose (Glu), fructose (Fru), and sucrose (Suc) and insoluble starch. NSC functions differ from that of structural carbohydrates: contrary to the latter, which are integrated into complex plant structures such as cell walls, NSCs are utilized not only as long- or short-term energy sources, via photosynthesis or respiration, but also as basic building blocks in several biosynthetic pathways [3].

A dynamic relationship is established in plants between carbon (C) fixed through photosynthesis and NSCs levels in plant tissues, predominantly in source leaves. Here, excess C fixed as Suc during the day, can be transported to photosynthetically inactive or inefficient sink tissues, to ensure their maintenance. Additionally, fixed C can be transiently stored as starch, mostly in the chloroplasts, in order to be reconverted to Suc during the night or in stress conditions that limit C assimilation, to sustain plant vitality [4]. Testimony to its metabolic ubiquity, Suc can also act as a feedback regulator of photosynthetic activity and of sink-source relationships in plants. In such manner, Suc levels may determine phenologic variation of growth patterns, trigger negative feed-back loops that limit C assimilation rates and C availability for growth and development [5]. The above processes are central for the resolution of the constant conundrum faced by plants growing in a continuously changing environment, in which they must decide between employing costly C resources for growth and reproduction, or for survival under usually multiple, perduring and simultaneous (a)biotic stress conditions. This decision is also dependent on additional endogenous factors related to the leaf´s ability to utilize luminous energy, such as age, morphology, weight and/ or anatomy, chlorophyll levels and sugar content [6–8].

Similarly to NSCs, proteins have a crucial metabolic function, catalyzing key reactions or acting as intermediaries in vital plant processes such as photosynthesis, respiration and concomitant electron transport, C and nitrogen (N) metabolism, response to stress and protective detoxification reactions and defense to biotic aggressors. Moreover, proteins involved in C- and N-related biochemical pathways usually show a greater variation with stress than NSCs [9, 10].

Although data regarding variations of primary and secondary metabolism in plants under natural conditions in the field are limited, they have nevertheless shown that both vary significantly in response to phenology and to changing environmental conditions [3, 11]. For instance, factors such as soil fertility, ambient temperature, water and CO_2_ availability are known to influence photosynthetic capacity and chlorophyll biosynthesis or breakdown, as well as related leaf metabolic pathways. These, in turn, will determine the distribution of species in different habitats and having diverse adaptive strategies [3, 12–14]. The ample climatic heterogeneity found in Mexico contributes to a high diversity of these determining factors, which can therefore sustain high rates of biological diversity. This is principally true regarding the flora of temperate and semi-humid regions [15]. The latter offer an ideal ambience to study the effect of environmental stress on the physiological responses of a large assortment of contrasting plant species.

The Ebenaceae plant family, which includes the *Diospyros* genus, is ubiquitously present in various physiographic regions of Mexico. Members of the latter, constituted by more than 20 arboreal and shrub species, grow at low population densities [16]. At least eight species are localized in tropical and temperate forests, shrubs and grasslands in a region in Western Mexico that includes the states of Michoacán and Jalisco. Particularly abundant in an ample location known as “Depresión del Balsas” localized in Jalisco are the perennial *D*. *digyna* trees regionally recognized as “Zapote negro”, which are highly appreciated for their berry fruits. On the other hand, the closely related deciduous *D*. *rekoi* trees, known as “zapotillo negro”, are restricted to a section of the trans-Mexican volcanic belt that also traverses the state of Jalisco. The fruit of these two species have been predominantly consumed by the local population for centuries, although variable increases in demand for larger markets are frequent, particularly for “zapote negro”. Despite their dietary importance, little is known regarding agronomical and phytochemical aspects of these species, which are considered to have a high marketing potential, mostly due to the nutraceutical and pharmacological properties attributed to the fruits, which are rich in triterpenoids, including steroids, and naphthoquinones [17]. The study herewith described represents an effort to gain a deeper understanding of the dynamics of NSCs, protein and chlorophyll variations in these two woody species in different seasons, which are hypothesized to be determined by their natural growing habitats, agricultural management practices and physiological plasticity. The effects of the latter on leaf secondary metabolism were also explored. In consequence, these variables were studied in the context of two different forest habitats with contrasting soil and climatic conditions.

## Materials and methods

### Plant material

Plant leaf samples were obtained in two different locations in Western Mexico. Sampling of perennial *D*. *digyna* (Ddg) trees was performed in an agro-forestry settlement in the municipality of Taretan, Michoacán (19° 20´ 00” N, 101° 55´ 00” W). This site, localized at 1100 meters above sea level (masl), and has a mean annual temperature and rainfall of 22°C and 1100 mm, respectively. On the other hand, the sampling of deciduous *D*. *rekoi* (Dre) trees was established based on previous collections of this species preserved in the IBUG-UDG herbarium of the University of Guadalajara, which led to the selection of the municipality of Teocuitatlán de Corona, Jalisco (20° 11´ 8” N, 103° 29´ 55” W) as the study site. The latter is localized at 1800 masl, and has a mean annual temperature and rainfall of 20°C and 531 mm, respectively. Both experimental sites are located in privately owned land. Verbal permission to conduct the study in these lands was awarded by the respective owners. Both *Diosypyros* species are not considered to be endangered species nor are they protected.

Five trees were randomly sampled in each sampling site. Sampled trees were 10 to 12 m high, with mean tree trunk diameters at chest-height (DCH) of 40 and 25 cm for Ddg and Dre, respectively. Mature leaves orientated to the four cardinal points and localized at mean height of the tree canopies, were sampled individually from each tree. Leaves were sampled in the morning, at the mid-point of every season, between winter of 2014–2015 and winter of 2015–2016. Ambient temperatures and relative humidity, measured with a digital hygrometer (TFA Dostmann GmbH & Co. KG, Wertheim, Germany) were determined simultaneously with leaf sampling. In addition, photosynthetic photon flux density (PPFD) was determined using a UV-Visible Li-Cor LI-250 quantum sensor (LI-COR, Lincoln, Nebraska USA). Additionally, these variables were constantly measured in a daily basis during the sampling period using meteorological data provided by the weather stations manned by The National Water Commission (Conagua; Mexico) which were localized in the vicinity of the sampling sites.

Leaf samples were kept in solid CO_2_ for transportation to their final destination, where they were subsequently stored at -20°C. Frozen fresh tissues were subsequently employed for chlorophyll quantification assays and phenolic acids identification, whereas lyophilized tissues were employed for NSCs, invertase activity and protein analysis (see below).

### Chlorophyll determination

Fresh leaf tissues were extracted with cold 80% acetone according to [18]. Total chlorophyll levels, as well as those of chlorophyll A (Chl a) and B (Chl b), were determined as described in [19], using a UV-visible spectrophotometer (LABOMED Inc., Los Angeles, CA, USA).

### Determination of non-structural carbohydrates (NSCs)

Leaf NSC levels were determined according to described methodologies [20, 21], with minor modifications. Briefly, lyophilized ground leaf tissues (25 mg) were mixed with 100 μl 80% v/ v aqueous ethanol, 100 mM Hepes buffer, pH 7.4, and 5 mM MgCl_2_ and incubated at 4°C for 10 min with stirring. After refrigerated centrifugation at 10,000 × g (4°C for 10 min), the cleared supernatants were transferred into fresh tubes and concentrated by vacuum centrifugation (Heto Maxi Dry Lyo, Heto-Holten, Denmark). The resulting pellets were re-dissolved in 100 μl of 100 mM Hepes buffer, pH 7.4, and 5 mM MgCl_2_, and were used for the determination of starch. Suc, Glu, and Fru were measured using enzyme-based methods, as instructed (Boehringer Mannheim/R-Biopharm, Darmstadt,Germany), except that the final reaction volume was reduced to fit a micro-plate format (250 μl per reaction). Starch levels were determined after α-amylase digestion of the insoluble pellets as described [22].

### *In vitro* determination of invertase and sucrose synthase and activities

Acid soluble (vacuolar), acid insoluble (cell wall), and neutral (cytoplasmic) invertase and sucrose synthase activities were determined according to described procedures [21, 23].

### Protein determination

Soluble leaf protein levels were determined according to the Bradford dye-binding method [24], with a commercial kit (Bio-Rad, Hercules, CA, USA) using bovine serum albumin as a standard.

### Thin layer chromatography of leaf extracts

Powdered lyophilized leaf tissues of both species (50 mg) were suspended in 1 ml of 60% MeOH and stirred for 24 h at 200 rpm 25°C and filtered. Band-wise application of the resulting leaf extracts (i.e., 2.5 μl) on 20 × 10 cm HPTLC silica gel 60 F254 plates (Merck KGaA, Darmstadt, Germany), was performed using a CAMAG automatic TLC sampler (ATS4) (CAMAG Chemie-Erzeugnisse & Adsorptionstechnik AG, Muttenz, Switzerland). A total of 20 tracks, 8.0 mm wide and 15.0 mm away from the lateral and lower edges, were run in each plate. The plates were run with a 20: 2: 2: 4 ethyl acetate–formic acid–acetic acid–water mobile phase in a CAMAG automatic developing chamber (ADC2) previously saturated for 20 min with a KSCN solution, and conditioned at 47% relative humidity for 10 min. For post-chromatographic derivatization, the plates were heated at 100°C for 3 min and, while still hot, immersed into a “Natural Products A” reagent (Carl Roth GmbH + Co. KG; Karlsruhe, Germany), dried and subsequently dipped into a polyethylene glycol 400 solution. In both cases, immersion speed and duration were 3 cm/ s and 3 s, respectively. Documentation was performed with a CAMAG TLC visualizer under white light and at UV light (at 366 nm) prior to, and after, derivatization.

### Physicochemical characterization of the soil

Soil samples surrounding each of the trees examined in this study were procured. In all cases, care was taken to ensure that all soil samples were taken around the rhizospheric zone of influence. The physicochemical analyses of the soil samples were performed according to the Mexican official standard 021-SEMARNAT [25].

### Data analysis

Mean total chlorophyll, chlorophyll a/ b ratios Chla/ Chlb), NSCs and protein content and invertases activity variables were analyzed with one-way ANOVAs to evaluate sources of variation resulting from the individual tree specimens and season by means of *a posteriori* Tukey test. In addition, a multiple linear regression analysis was performed to determine the relationship between Chla/ Chlb and PPFD. All tests were performed using the R package version 3.2.2 (http://www.rproject.org) with *a* ≤ 0.05 significance level.

A correlation matrix was generated using a parametric Pearson test. The results were plotted using RStudio package, in which the intensities of the blue and red dots represented positive and negative correlations, respectively. To evaluate the variance of the data, a multivariate principal component analysis (PCA) was conducted. A Hellinguer standardization of biochemical data was performed and mean eigenvalues were calculated to generate the axes using the Kaiser-Guttman criterion. The vectors in the graphs were generated by calculating de multiple regression variables with the above axes. The significance was calculated by means of permutations (999) employing a Bonferroni adjustment for multiple comparisons.

## Results

Ddg trees sampled in Taretan were associated with a sub-deciduous tropical forest. This species was abundant in this region, where 20 m-high trees having tree trunk diameters at DCH exceeding 70 cm were common. In contrast, the Dre population sampled in Teocuitatlán de Corona were located in a deciduous tropical forest that mingled with shrub sections. Dre trees were scarce, reaching maximum heights of 15 m and tree trunks with DCH ≤ 40 cm.

**Fig 1:**
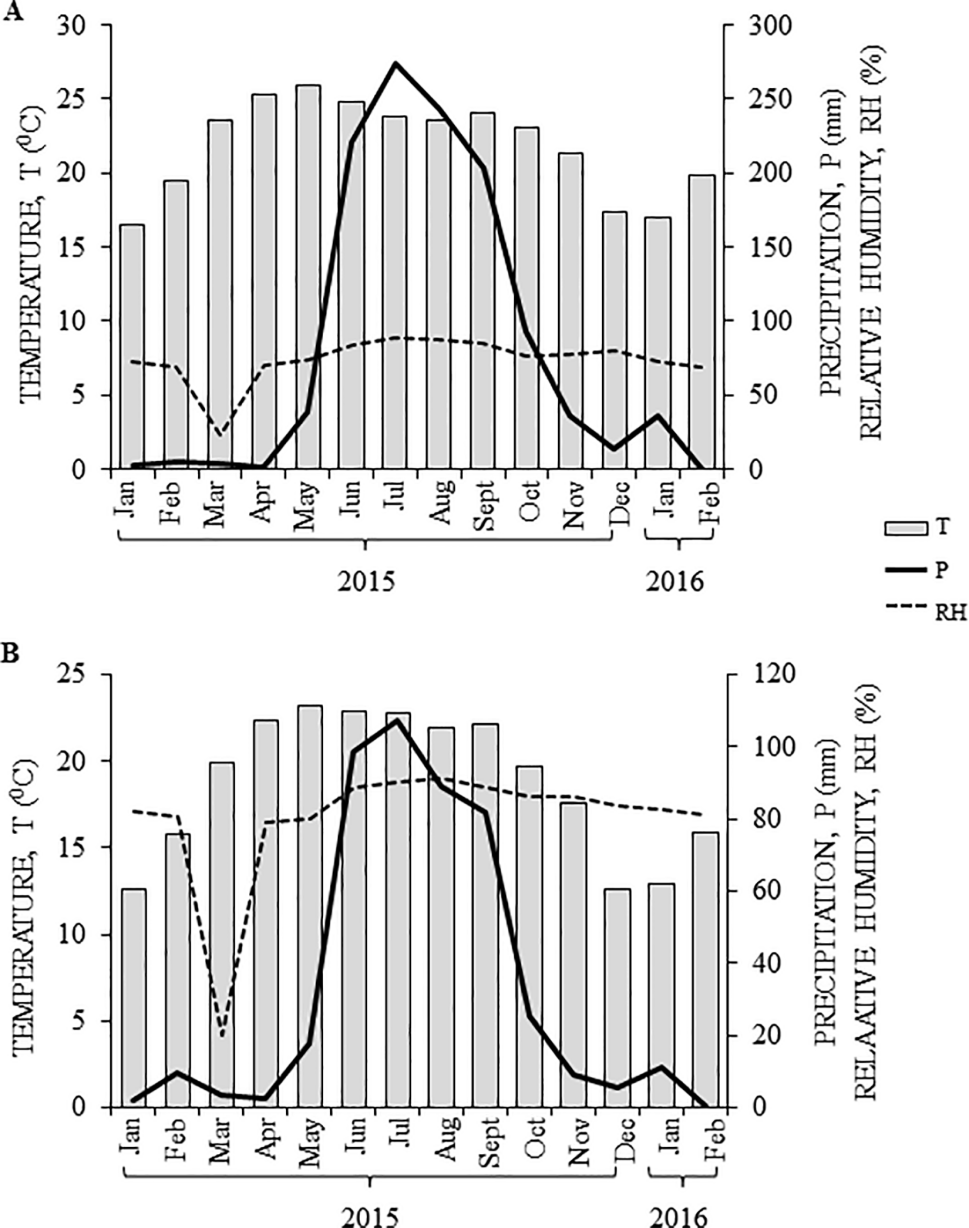
Climatic conditions in *Diospyros* sampling sites. Monthly average temperature (T, gray bars), relative humidity (RH, discontinuous lines) and precipitation (P, continuous lines) for the winter 2014-15-winter 2015–16 period, recorded at the (**A**) Taretan, Michoacán and (**B**) Teocuitatlán de Corona, Jalisco, Mexico, sampling sites for *D*. *digyna* and *D*. *rekoi*, respectively.

Climatological parameters recorded during the sampling periods in Taretan and Teocuitatlán de Corona are shown in Fig 1. Atypical results were obtained in both sites; in the former, annual rainfall was 70 mm higher and mean temperatures were 2°C lower than the historical climate normality for the site (Fig 1A). Conversely, annual rainfall and temperatures registered in Teocuitatlán de Corona were 40 mm lower and 3°C higher than the historical climate normality for the site (Fig 1B). Average PPFD was highly variable between trees, seasons and study sites (see below). Very similar average seasonal solar irradiance on tree horizontal surfaces were recorded in the two study sites for the duration of the study (S1 and S2 Tables). In both, highest values were recorded in the spring and summer of 2015, and lowest in the winter, particularly in 2014–15, as expected. However, PPFD monitoring of individual trees yielded a different scenario. Thus, seasonal PPFD spot testing in Taretan indicated that PPFDs were consistently low for T4 and T5 trees, being 10 to 15-fold lower than the PPFD received by the other trees in the spring-autumn of 2015, irrespective of the season (S3 Table). In addition, except for T1, PPFDs were lowest in the winter of 2015–16. In contrast, and coinciding with the seasonal solar irradiance, the highest PPFDs were recorded, either in the summer (i.e., T1 and T3 trees), or the autumn (i.e., T3 tree), of 2015. On the other hand, PPFDs recorded in Teocuitatlán de Corona were higher, in average, than those recorded in Taretan (S4 Table). In the former site, the lowest PPFDs for all trees were recorded in the summer and autumn of 2015. In addition, unusually high PPFDs were recorded in tree T2 in both winter seasons, whereas the highest average PPFDs were detected in the winter 2014-15-spring 2015 and in the winter of 2015–16.

The physicochemical parameters of the soil samples taken from both study sites are shown in Table 1. The variation between the soil samples taken around the five trees analyzed was high in Taretan, whereas the soil samples in Teocuitatlán de Corona were more homogenous, and could be separated into two well-defined groups according to their composition (Table 1).

**Table 1:** Soil physicochemical parameters of soil samples taken from the rhizospheric zone of influence around five (T1-to-T5) *Diospyros digyna* and *D*. *rekoi* trees sampled in Teocuitatlán, Jal., Mexico, and Taretan, Mich., Mexico, respectively.

	*Teocuitatlán, Jal.		Taretan, Mich				
Parameter	T1, T4, T5	T3, T2	T1	T2	T3	T4	T5
Density [ρ] (g/ cm^3^)	2.25	2.14	2.42	2.45	2.33	2.39	2.38
Textural class	^1^LS	LS	^2^SCL	SCL	SCL	^3^SL	SCL
**σ (ohms/ cm)	0.17	0.29	0.1	0.1	0.12	0.11	0.12
***CEC (meq/ 100 g)	66.85	87.85	44.1	47.6	46.55	42	53.2
pH	6.33	6.85	6.11	6.45	6.37	6.38	6.43
Organic matter (%)	10.31	8.88	2.58	4.98	2.37	5.11	6.8
N total (%)	0.44	0.86	0.2	0.16	0.17	0.28	0.22
P (ppm)	25	50	25	25	50	25	25
K (ppm)	180	250	250	180	180	180	120

*Mean values (n = 6)

**electrical conductivity

***CEC = cation exchange capacity

^1^LS = loamy sand

^2^ SCL = sandy clay loam

^3^SL = sandy loam

**Fig 2:**
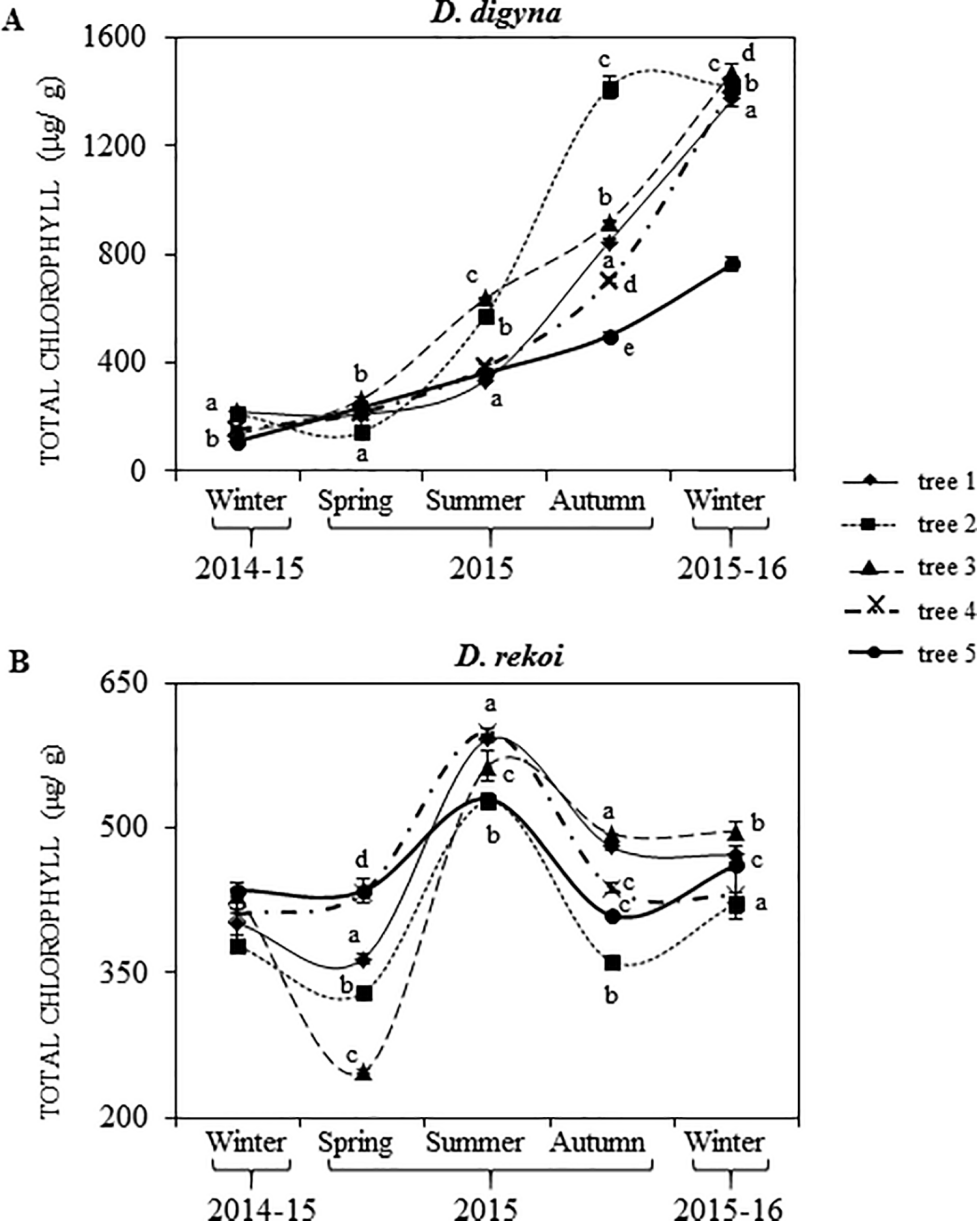
Seasonal variation in total chlorophyll (C_T_). C_T_ was determined in leaves of (**A**) *Diospyros digyna* or (**B**) *D*. *rekoi* trees for the winter 2014-15-winter 2015–16 period. A total of 5 trees per species were sampled throughout this period, each of which is represented by a different symbol. Each symbol represents the mean value of each determination (n = 12). Intervals over the symbols represent the standard error of the means, whereas different letters over the symbols represent statistically significant differences at *p* ≤ 0.05 (Tukey-Kramer test).

Total chlorophyll (C_T_) contents determined seasonally in leaves of Ddg and Dre are shown in Fig 2A and 2B, respectively. C_T_ contents gradually increased in leaves of Ddg trees during the period of experimentation, being highest in the winter of 2015. Moreover, significant differences were detected between the C_T_ content of the leaves of several Ddg trees sampled, predominantly in the autumn of 2015 (Fig 2A).

Regarding Dre, significant differences in leaf C_T_ levels were similarly observed, both between tree specimens and between seasons, except in the winter 2014–15 (Fig 2B). The summer season was characterized by the highest leaf C_T_ levels recorded. High average C_T_ contents in leaves of trees sampled in the summer and autumn of 2015, together with the lower average leaf C_T_ content in trees sampled in the winter 2014–15 and in the spring of 2015, inversely coincided with the PPFDs recorded in these seasons (S4 Table). A similar inverse correlation between leaf C_T_ content and PPFDs (S3 Table) was observed in Ddg trees sampled in Taretan from summer to winter of 2015.

The Chl a/ Chl b ratio data are shown in Table 2. The seasonal variations in Chl a/ Chl b ratios were more evident in leaves of Dre trees, particularly in the transition from spring to summer of 2015 when the Chl a/ Chl b ratios decreased significantly in all the trees examined. Chl a/ Chl b ratios subsequently increased in leaves of trees sampled, except in tree T3, in the fall of 2015 and remained mostly unchanged thereafter. Conversely, seasonal Chl a/ Chl b ratio variation in Ddg was less noticeable, although a tendency toward higher Chl a/ Chl b ratios was observed in the autumn-winter of 2015. Also noticeable was the T5 specimen, which showed a significant and consistent increase in this parameter from the winter of 2014 onwards. Conversely, Chl a/ Chl b ratios remained unchanged in tree T1 through the duration of the experiment. A poor and sometimes contradictory correlation was found between Chl a/ Chl b ratios and PPFD for both species (S5 and S6 Tables).

**Table 2:** Chlorophyll a/ chlorophyll b (Chla/ Chlb) ratios in leaves of five (T1- to-T5) *Diospyros digyna* and *D*. *rekoi* trees for the winter 2014-15-winter 2015–16 period.

		Chla / Chlb									
		*D*. *digyna*					*D*. *rekoi*				
		T1	T2	T3	T4	T5	T1	T2	T3	T4	T5
**2014**	Winter	*0.805 ^a^	0.825 ^a^	0.695 ^a^	0.722 ^a^	0.623 ^a^	0.754 ^a^	1.060 ^a^	0.647 ^a^	0.684 ^a^	0.846 ^a^
	Spring	0.866 ^a^	0.774 ^a^	0.816 ^a^	0.850 ^a^	0.853 ^b^	1.068 ^a^	0.693 ^b^	0.919 ^b^	0.885 ^b^	0.918 ^c^
	Summer	0.838 ^a^	0.391 ^b^	0.258 ^b^	0.838 ^a^	0.855 ^b^	0.568 ^b^	0.569 ^c^	0.555 ^a^	0.567 ^c^	0.554 ^b^
	Autumn	0.935 ^a^	0.851 ^a^	1.010 ^c^	1.023 ^b^	0.839 ^b^	0.718 ^b c^	1.273 ^d^	0.595 ^a^	0.735 ^b^	0.876 ^a^
**2015**	Winter	0.918 ^a^	0.904 ^a^	0.916 ^a c^	0.860 ^a^	1.090 ^c^	0.718 ^c^	0.938 ^a^	0.636 ^a^	0.821 ^b^	0.603 ^b^

*Mean values (n = 12); different letters indicate statistically significant differences at *p* < 0.05 (Tukey-Kramer test)

**Fig 3:**
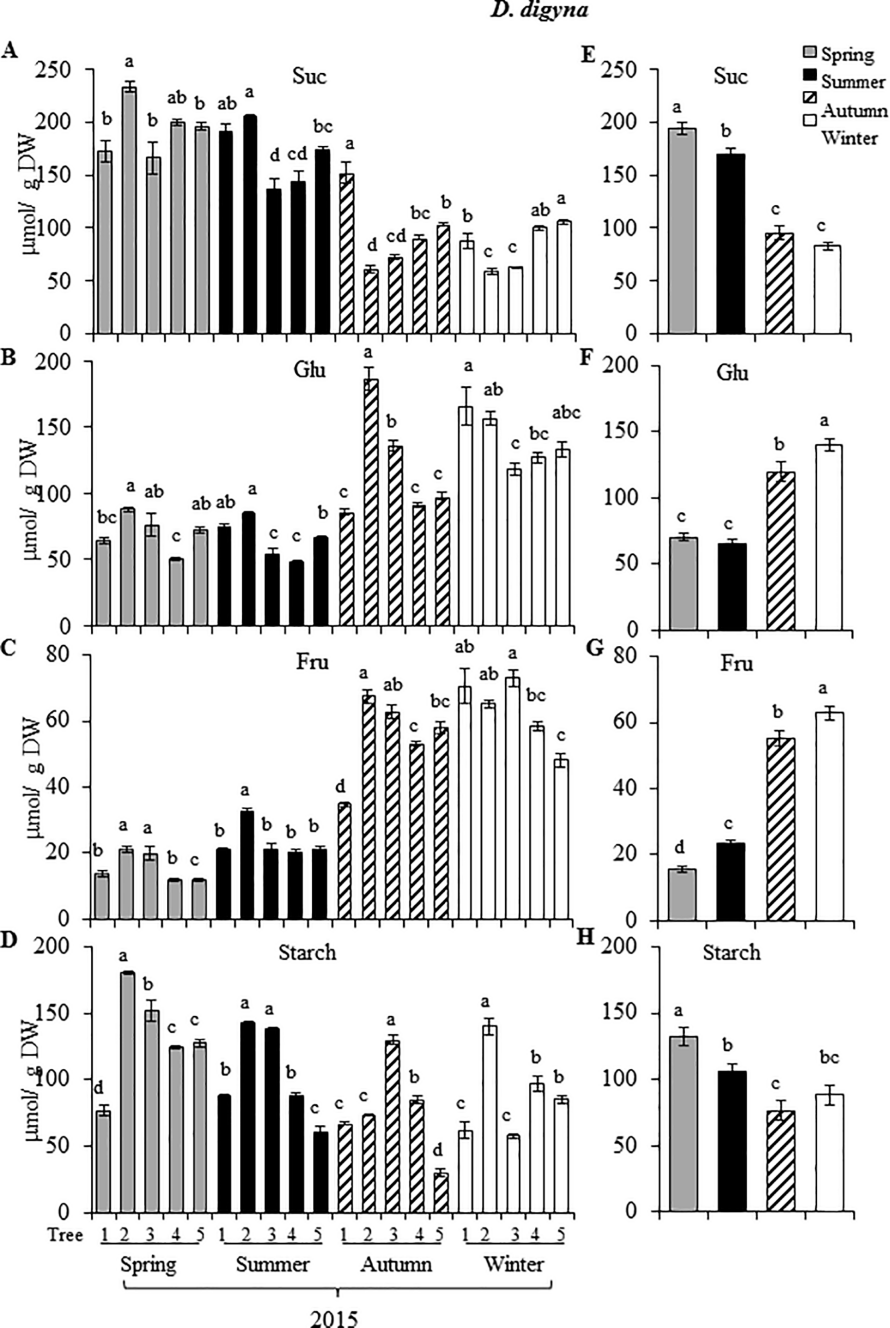
Seasonal variation in soluble and insoluble non-structural carbohydrates in *Diospyros digyna*. Levels of (**A**) sucrose, (**B**) glucose, (**C**) fructose, and (**D**) starch in leaves of 5 individual *D*. *digyna* trees (1–5) for the year 2015. The bars represent mean values of each determination (n = 10). Average seasonal variation in each respective non-structural carbohydrate analyzed is shown in (**E**) to (**H**). Intervals over the bars represent the standard error of the means, whereas different letters over the bars represent statistically different values at *p* ≤ 0.05 (Tukey Kramer test). DW = dry weight.

Soluble NSC accumulation in leaves of Ddg was clearly influenced by the season of the year. Thus, in 2015, foliar Glu and Fru levels increased from minimal values in the spring to maximum levels in the winter (Fig 3B and 3F, and Fig 3C and 3G, respectively). An inverse pattern was observed for Suc, whose levels reached highest levels in spring and lowest in winter of 2015 (Fig 3A and 3E). Contrary to soluble NSCs, starch levels did not vary in a discernible pattern, since a high seasonal variability in starch levels was detected between individual trees (Fig 3D). However, a tendency toward lower foliar starch levels as the year progressed from spring to winter, similar but less distinct than that of Suc, was observed (Fig 3H).

**Fig 4:**
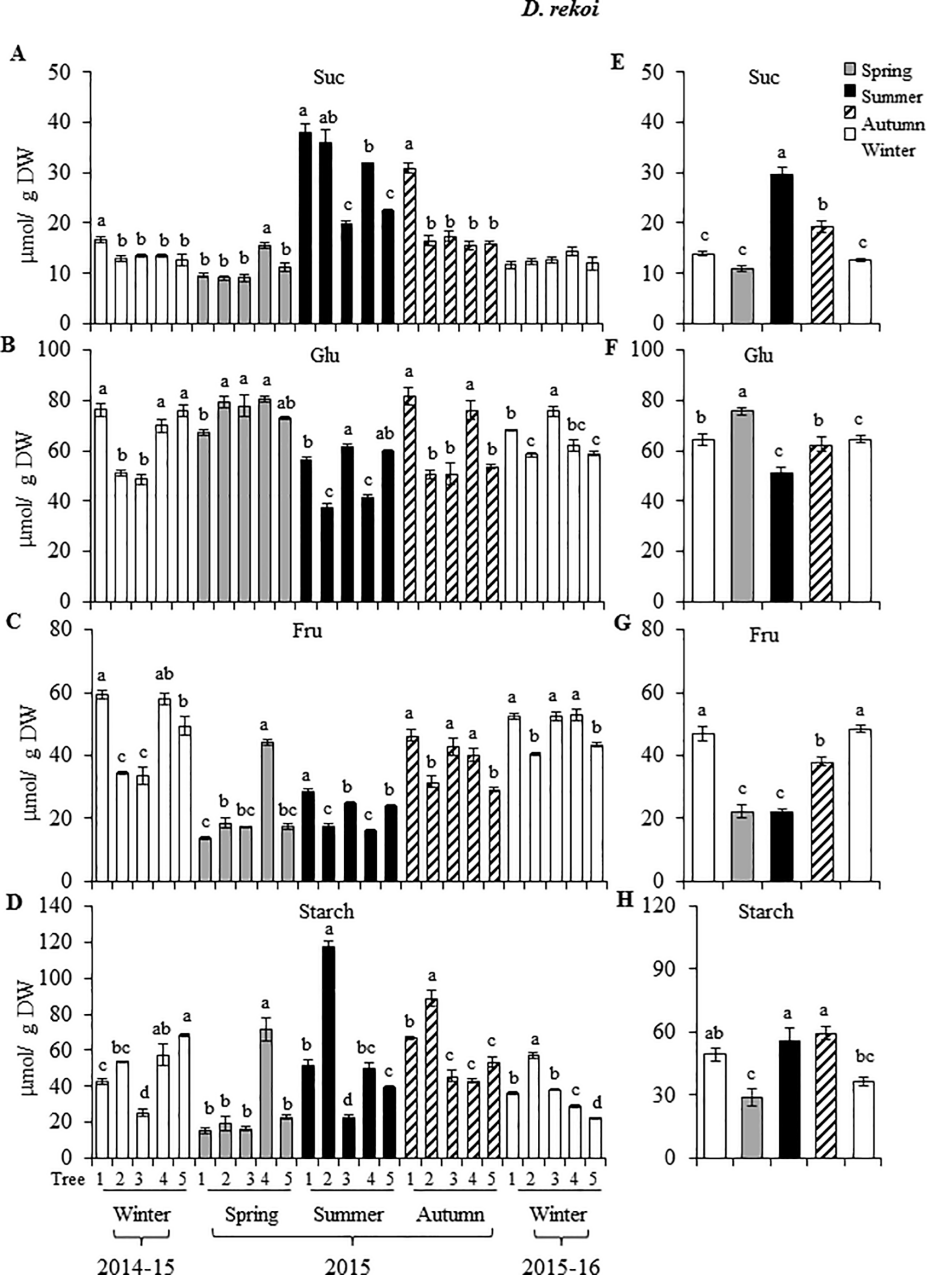
Seasonal variation in soluble and insoluble non-structural carbohydrates in Diospyros rekoi. Levels of (A) sucrose, (B) glucose, (C) fructose, and (D) starch in leaves of 5 individual D. rekoi trees (1–5) for the winter 2014-15-winter 2015–16 period. The bars represent mean values of each determination (n = 10). Average seasonal variation in each respective non-structural carbohydrate analyzed is shown in (E) to (H). Intervals over the bars represent the standard error of the means, whereas different letters over the bars represent statistically different values at p ≤ 0.05 (Tukey Kramer test). DW = dry weight.

The seasonal variations in NSC contents in leaves of Dre (which were approximately 2-fold lower than those in leaves of Ddg) were less obvious, due to significant variation detected between specimens, within and between seasons (Fig 4). Despite this disparity, the pattern observed was different from that observed in leaves of Ddg. Thus, Suc, and to a lesser degree, starch, tended to peak in the summer and spring, whereas relatively constantly low levels of these NSCs were detected in all other seasons (Fig 4A and 4E, and Fig 4D and 4H, respectively). In contrast, Glu and Fru tended to decline to their lowest levels in the summer, down from relatively constant levels in all other seasons (Fig 4B and 4F and Fig 4C and 4G, respectively).

**Fig 5:**
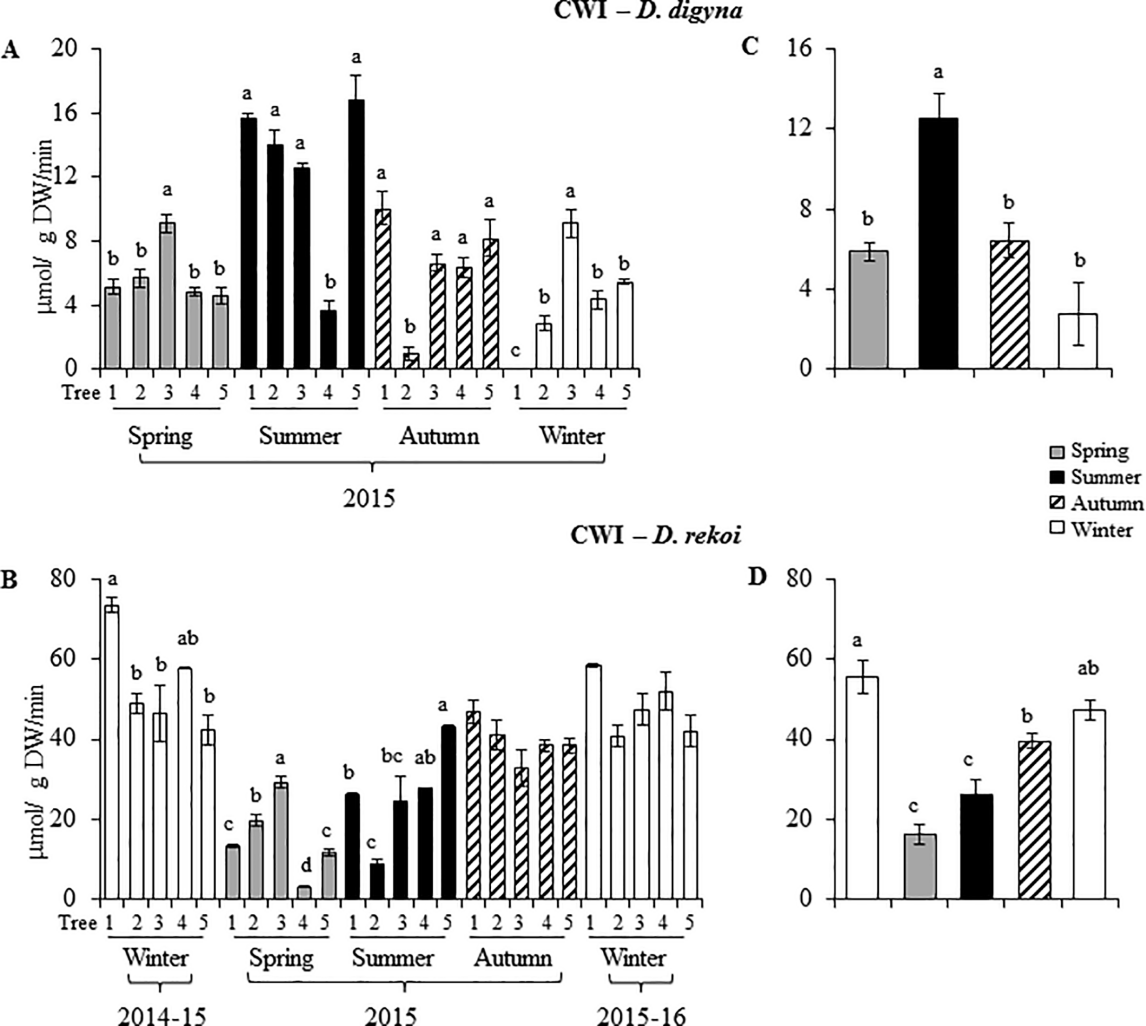
Seasonal variation in cell wall invertase activity (CWI) in *Diospyros digyna* and *D*. *rekoi*. CWI activity levels were determined in leaves of five (1–5) (**A**) *Diospyros digyna* and (**B**) *D*. *rekoi* trees for the year 2015 (*D*. *digyna*), or for the winter 2014-15-winter 2015–16 period (*D*. *rekoi*). The bars represent mean values of each determination (n = 10). Average seasonal variation in CWI activity in (**C**) *D*. *digyna* and (**D**) *D*. *rekoi* are also shown. Intervals over the bars represent the standard error of the means, whereas different letters over the bars represent statistically different values at p ≤ 0.05 (Tukey Kramer test). DW = dry weight.

Leaf sucrose synthase (SuSy) and invertase activities were determined in both species to define their contribution to the soluble NSC seasonal fluctuations observed. Cell wall invertase (CWI) activity was clearly predominant in both species (Fig 5), with ca. 4-to5-fold higher activities found in leaves of Dre (Fig 5B and 5D). Apart from a few exceptions, leaf CWI activity did not vary among the trees specimens sampled each season. However, significant seasonal variations were observed. Thus, CWI activity was highest in leaves of Ddg trees sampled in the summer of 2015, and lowest in the winter of the same year (Fig 5A and 5C). In contrast, CWI activity in leaves of Dre was low in the spring and summer of 2015 and high in both winter seasons sampled (Fig 5B and 5D). Only in the latter case did leaf soluble NSC accumulation patterns coincide with CWI activity.

Other invertase activities were detected in *Diospyros* trees. Although they showed clearly defined seasonal patterns of activity, these did not coincide with the NSC level fluctuations observed. In addition, variation detected between trees was considerable, due perhaps to technical difficulties. Thus, neither vacuolar invertase (VI) nor neutral/ alkaline cytoplasmic invertase (CI) activities were considered to be experimentally valid and are, therefore, not shown. Conversely, no SuSy activity was detected in leaves of Ddg and Dre trees.

**Fig 6:**
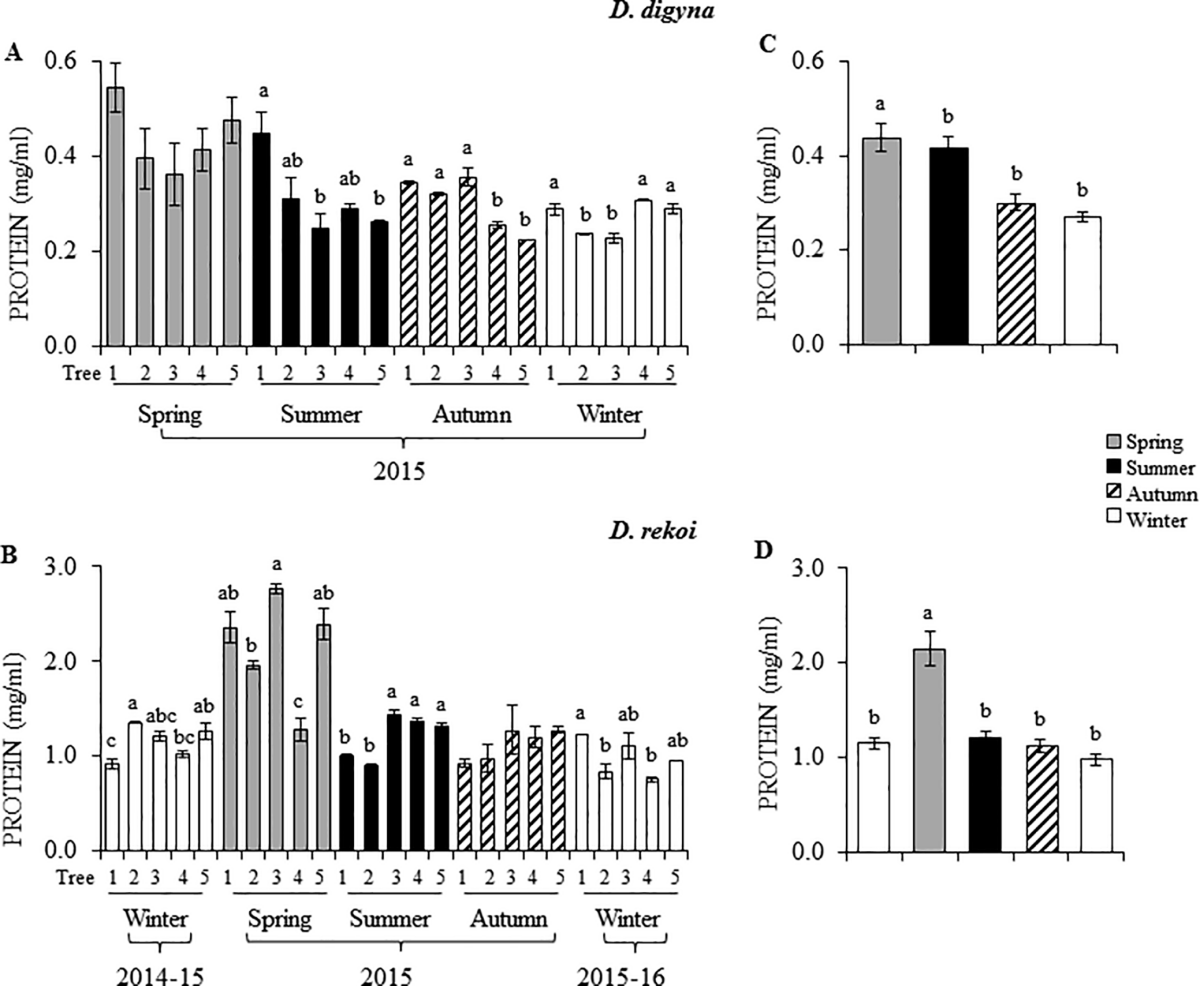
Seasonal variation in protein content in *Diospyros digyna* and *D*. *rekoi*. Foliar protein levels were determined in five (1–5) (**A**) *D*. *digyna* and (**B**) *D*. *rekoi* trees for the year 2015 (*D*. *digyna*), or for the winter 2014-15-winter 2015–16 period (*D*. *rekoi*). The bars represent mean values of each determination (n = 6). Average seasonal variation in leaf protein content in (**C**) *D*. *digyna* and (**D**) *D*. *rekoi* are also shown. Intervals over the bars represent the standard error of the means, whereas different letters over the bars represent statistically different values at *p* ≤ 0.05 (Tukey Kramer test). DW = dry weight.

Compared to Ddg, protein content in leaves of Dre was 4- to 5-fold higher (Fig 6). However, seasonal changes in leaf protein levels were similar in both species, reaching the highest protein levels in the spring (Fig 6A–6D).

**Fig 7:**
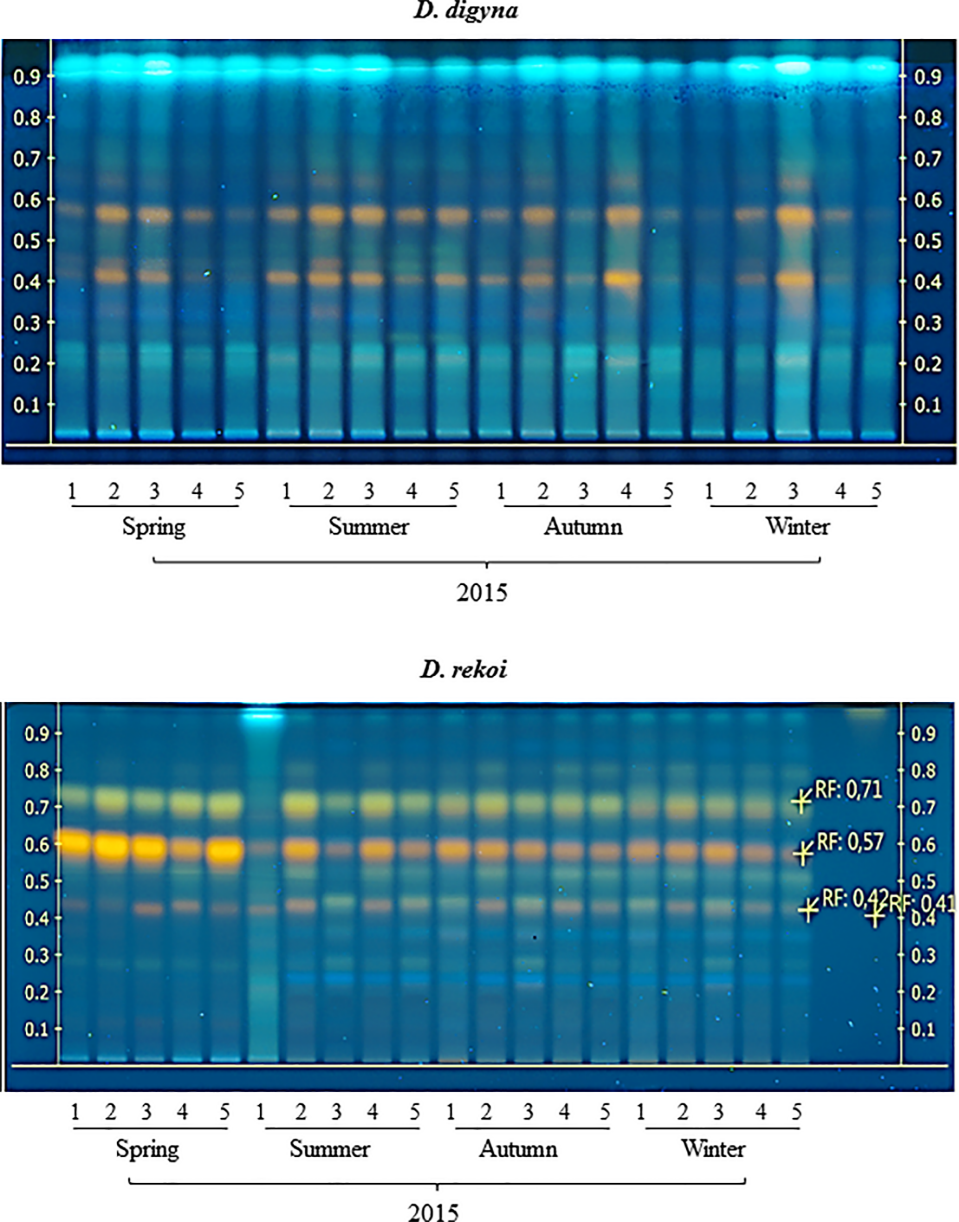
Seasonal variation in phenolic acid content in *Diospyros digyna* and *D*. *rekoi*. Phenolic acids TLC traces of (**A**) *D*. *digyna* and (**B**) *D*. *rekoi* leaf extracts are visualized under UV light (366 nm) after derivatization with the NP/ PEG reagent (see materials and methods). Lanes: 1–5 extracts from trees 1–5 (spring 2015); lanes 6–10, extracts from trees 1–5 (summer 2015); lanes 11–15, extracts from trees 1–5 (autumn 2015), and lanes 16–20, extracts from trees 1 to 5 (winter 2015). The Rf values of the most prominent bands are included in the TLC trace in (**B**).

Likewise, the results of the TLC determination of total phenolic acids in leaves of Ddg and Dre, shown in Fig 7, indicate that these compounds were particularly abundant in leaves of Dre sampled in the spring season (Fig 7B), In contrast, a lower accumulation of these compounds, which showed no seasonal variation, was detected leaves of Ddg (Fig 7A). The precise chemical constitution of these compounds has yet to be determined, although preliminary evidence suggests that they are mostly flavones.

Finally, the results obtained from the correlation analyses performed with all experimental parameters measured in this study (S1 and S2 Figs) indicate that C_T_ and Suc explained 22% and 28% of the biochemical variation observed in Ddg. On the other, these were mainly influenced by three clearly dominant environmental factors, such as temperature (T), relative humidity (RH) and solar radiation (R). Factors found to have a lesser influence were the organic matter (OM), N and P content of the soil, in addition to its pH value (S3 Fig). In contrast to Ddg, the parameters that better explained the biochemical variation in Dre were protein (28%) and starch (26%). Temperature (T) and relative humidity (RH) remained as the principal environmental factors affecting the biochemical variation in Dre. However, in contrast to Ddg, the effect of R was diminished. In Dre, this factor had a minor influence, similar to that of K, pH, N, P and OM of the soil (S4 Fig).

## Discussion

In this study, deciduous and perennial *Diospyros* tree species were located in plantations cultivated under clearly contrasting conditions and were sampled in the course of a 16-month period (winter 2014–15 to winter 2015–16). As shown in Fig 1, Ddg trees sampled in Taretan were grown in a dry ambience, despite having higher annual average temperatures and rainfall. Conversely, Dre trees grew in a more humid and temperate site, regardless of the lower rainfall received. The higher humidity recorded was probably a result of the topography the site, located in a ravine with an N-S orientation. Likewise, data in Table 1 indicate that the study sites had differences in most other important parameters, such as those defining soil texture and nutrients. Thus, soil in Teocuitatlán de Corona was lighter, slightly less acid, and had a much higher nutritional quality in terms of soil conductivity, cationic ion exchange capacity, organic material and N content. The latter could reflect the fact that this was an unperturbed site, where trees grew in the wild. This panorama contrasted with Taretan, where the cultivation of semi-domesticated Ddg trees was more intensive. Hence, the differing climatic and soil conditions encountered in both sites probably contributed, at least partially, to the physicochemical differences detected in the foliage of both tree species during the period of study (see below).

For instance, although seasonal C_T_ variations were detected in both *Diospyros* species, the C_T_ content in Ddg leaves was consistently higher, irrespective of the season in which it was measured (Fig 2). This tendency agreed with differences in leaf chlorophyll content per unit leaf area usually observed between leaves of tropical evergreen trees and temperate deciduous trees, which are generally lower in the latter [26]. In this respect, the characteristics of Dre’s sampling site were in agreement with a recent panbiogoegraphic analysis of *Diospyros* spp. in Mexico that reported the presence of Dre exclusively in deciduous forests [17].

Another difference observed was the positive association between leaf C_T_ content and rainfall (compare Figs 1B and 2B) in Dre trees. In these, foliar C_T_ values reached maximum values during the rainy season in the summer and returned to basal C_T_ levels in the subsequent fall-winter period. In like manner, C_T_ fluctuation in leaves of Dre trees inversely correlated with the average PPFDs (S4 Table) and the Chl a/ Chl b ratios (Table 2) recorded, particularly in the summer, when these parameters were at, or close to, their lower limits. Similar findings were reported in a study involving Bignoniaceae tropical woody species, in which Chl a/ Chl b ratios consistently showed a negatively correlation with Chl contents across light treatments [27]. In contrast, foliar C_T_ content in Ddg trees was found to gradually increase as the year progressed (Fig 2A), reaching the highest accumulation in the winter of 2015, in clear contrast with those recorded in the previous winter season, when C_T_ values were their lowest. Additionally, C_T_ values did not inversely correlate with the Chl a/ Chl b ratios (Table 2), as observed in Dre. This behavior is difficult to explain since it contrasted with the usual reduction in C_T_ usually observed in woody species during the summer to fall-winter transition. It also contradicted findings of a related study showing that C_T_ reached minimal or drastically reduced levels in the deciduous and perennial tree population of a temperate forest in Belgium during the winter [28]. However, one study indicated that chlorophyll a + b composition in leaves of cork oak (*Quercus suber*) can remain steady throughout the year, undergoing only temporarily limited seasonal changes [29], whereas another found that chlorophyll concentration variation in several tropical forest tree species growing under varying light conditions in Brunei was a highly plastic response [26]. In addition, mention can also be made that foliage acclimation to light is also dependent on the interactions between traits with strongly varying time kinetics, such as N leaf content, Chl/ carotenoid ratios and degree of fluorescence emission [30]. Thus, it may be argued that the unexpectedly high C_T_ values recorded in leaves of Ddg in the winter of 2015, together with the stark contrast in Chl a/ Chl b ratios observed between winter 2014 and winter 2015 (see below), could have been a reflection of other phenomena occurring in this growing site. For instance, PPFDs were, in average, the lowest in the winter 2015 (S3 Table), a condition that could have explained the un-seasonally high Ct values, and further interpreted as an acclimation response to shade designed to improve plant C gain by enhancing light interception [30, 31].

Fluctuations in Chl a/ Chl b ratios are considered to reflect the changes in the relation of PSII to PSI that occur in response to variations in irradiance. It is usually low under shade conditions due to the increase in Chl b content, principally in thylakoid light-harvesting complexes, required for the capture of more light to increase photosynthetic efficiency. On the other hand, higher Chl a/ Chl b ratios are believed to be produced under high irradiance as Chl b levels decrease in order to reduce light absorption and contribute to the photo-protection of the PSII complex [32–34]. As shown in Table 2, Chl a/ Chl b ratios in leaves of all Dre trees sampled were significantly at their lowest in the summer of 2015, whereas significantly minimal summer leaf Chl a/ Chl b ratios were detected only in two Ddg trees (i.e., T2 and T3). In Dre, this response was in accordance with the need to capture more light in trees exposed to low PPFDs. However, it held true for the summer of 2015 only, since the similarly low PPFDs recorded in the subsequent autumn season did not lead to a corresponding reduction in the Chl a/ Chl b ratios. Moreover, the low Chl a/ Chl b ratios detected in leaves of the T2 and T3 Ddg trees were not in agreement with the high PPFDs received by these trees in this season, being contrary to the expected high Chl a/ Chl b ratios needed as a photo-protective response under high illumination. In addition, the Chl a/ Chl b ratio in the Ddg T1 specimen remained constant, irrespective of seasonal variations in irradiation. In consequence, data in S5 and S6 Tables showed that PPFD received by the individual trees in both locations poorly correlated with the magnitude of their Chl a/ Chl b ratios.

This unexpected outcome suggests that factors other than light intensity were having an important influence on the foliar Chl a/ Chl b ratios in *Diospyros* trees. Among those previously reported to influence this parameter are variations in the spectral quality of the canopy light and its relation to the stoichiometry of pigment-binding protein complexes [35] and changes in the biosynthesis of photo-protective secondary metabolites such as carotenoids, flavonoids and/ or phenolic compounds [36]. Interestingly, flavonoid glucosides have been also suggested to function as reserve carbohydrate in evergreen lemon leaves [37] (see below). However, data shown in Fig 7, indicate that total phenolic content in leaves was clearly higher in Dre, manifestly in the spring of 2015. This result negated the possibility that the accumulation of foliar photo-protective metabolites in Ddg, possibly generated from a higher carbohydrate availability in leaves (see below), was a factor contributing to the unexpected Chl a/ Chl b ratios observed. On the other hand, the lack of phenolic acid accumulation in Ddg leaves was in agreement with a recent comparative study of juvenile evergreen and deciduous shrub species [38] in which, contrary to a prevailing hypothesis, phenolic compound levels were found to be lower in the longer-living leaves of evergreen species. Such pattern disproved the proposal that these secondary metabolites should accumulate to higher concentrations in these leaves, believed to be a major C storage site, at least at the juvenile stage, (in contrast deciduous trees where C storage occurs in woody branches, stems and roots) in order to protect them from insect herbivory. Thus, the above study concluded that evergreen plants may utilize structural modifications for defense against herbivores, rather than rely on the accumulation of secondary metabolites. This proposal was in agreement with the thicker and coarser leaf morphology of Ddg leaves (not shown) and with their low foliar phenolic acids content (Fig 7A)

In addition, differences in N content in leaves, represented as protein content (Fig 6) could have been responsible, at least partially, for the anomalous Chl a/ Chl b ratios detected in Ddg. For instance, the high Chl a/ Chl b ratios detected in the autumn and winter of 2015 (Table 2) coincided with the lowest leaf protein contents recorded. This reciprocity partly agreed with the predicted increase of the Chl a/ Chl b ratios in response to decreased leaf N contents previously proposed by a theoretical prediction of optimal N partitioning in leaves [27]. The latter is supported on the importance that leaf N has on important photosynthetic characteristics, such as maximum electron transport capacity and carboxylation rate [39]. On the other hand, the low Chl a/ Chl b ratios observed in the summer of 2015 in Dre coincided with the transient peak of Suc and starch leaf contents, which suggested that an efficient transfer of light energy to carbon accumulation may have also contributed to reduce photo-inhibitory effects and, consequently, the Chl a/ Chl b ratios. In this respect, it may be equivalently argued that the low degree of correlation between leaf Chl a/ Chl b ratios and irradiance observed in leaves of Ddg could be partially associated by the efficient transformation of light energy to NSCs, which were, in average, 2-fold higher than those detected in *D*. *rekoi* leaves (Figs 3 and 4). The observed difference occurred despite the fact that respiration, assumed to largely affect the C budget of plants, in combination with growth and maintenance, is exponentially related to temperature [40, 41], which was higher in the Ddg sampling site. Additionally, warmer soil and air temperatures usually accelerate the initiation of spring net primary production and delay autumn senescence [41]. Yet, the higher NSC accumulation in leaves of Ddg was contrary to several previous studies revealing that evergreen species, including arctic shrubs, Mediterranean and neo-tropical woody species and conifers, usually have lower NSC concentrations than deciduous species [38]. They agreed, however, with a handful of other reports in which lower NSC contents were detected in deciduous species, in comparison to evergreen [42] or semi-evergreen [43] tree species.

NSC leaf contents also showed evident seasonal fluctuations that were distinct for each species. Accordingly, Suc and starch accumulation in Ddg leaves showed a gradual decrease from maximum levels in the spring to minimal levels in the autumn 2015-winter of 2015–16. In comparison, Glu and Fru levels reached their highest levels in the autumn 2015-winter 2015–16. Suchlike NSC fluctuations can be explained as a need to support flowering in Ddg trees, which was unexpectedly observed in the autumn, posterior to the normal spring flowering season, and also by the extended fruit maturation process. Curiously, this anomalous behavior simulated that of subtropical fruit perennial species, such as the Loquat tree (*Eriobotrya japonica*) and other perennial fruit species, e.g. citrus trees [44, 45]. The latter usually rest in the summer, bloom in the autumn, and proceed to the fruit set, development and ripening stage in the winter and early spring, which concludes just before the emergence of new vegetative growth. Thus, the drop in Suc observed in the winter 2015 was possibly in accordance with the high demand for photosynthates that fruit growth represents, as has been determined in certain perennial fruit species, such as citrus trees [44]. In addition, the high Suc content that accumulated in the highly resource-demanding spring season, was in accordance with the fact that in active source organs sucroneogenesis dominates over sucrolysis [46]. On the other hand, the commonly observed wintry depletion of leaf starch levels was not as evident in the winter of 2015. This difference was perhaps indicative that, in contrast to deciduous trees which mostly store their reserves in woody tissues, leaves represent a C reservoir in perennial trees that only complements the major C storage present in the roots [47, 48] which is used to support mostly vegetative growth during the spring flush. Another possible factor related to lack of the expected starch reserve depletion observed in this season was the mentioned heterogeneous fruit set and development that was observed in all five Ddg trees (E. Ramírez-Briones, personal communication). Likewise, and similarly to other perennial fruit trees, fruit development in Ddg might have been sustained by the root C reserves [49–51]. This possibility remains to be demonstrated experimentally.

The seasonal NSC fluctuation pattern observed in Dre was contrary to the main period of NSC reserves accumulation of most temperate deciduous trees, which takes place in early autumn, although they partially agreed with the mass mobilization of these reserves that happens later in the same season [52]. They also coincided with the substantial NSC depletion in the spring usually observed in these trees, which is needed to support regrowth and with the subsequent buildup of reserves during the summer and early autumn [53]. Thus, in deciduous peach trees, C reserves drastically decrease between February and May, especially in the above-ground organs, as a consequence of the high sink strength represented by new organs at bud break. Later, and similarly to Dre, summer C reserves have been shown to increase in all tree reserve compartments, at least in peach [52]. A comparable pattern has also been observed in other deciduous trees, such as beech, *Populus tremuloides*, sugar maple and mature oaks, which can accumulate C reserves during the summer [52, 54]. However, mention should be made that low rates of NSC accumulation during summer or early autumn periods have also been reported in peach and other deciduous fruit trees during peak fruit growth [52, 55, 56]. Based on the above, it could be argued that, in general, the seasonal NCS content in Dre agreed with the typical pattern observed in temperate deciduous trees. In these, tissue NSC concentrations usually fall during winter dormancy, early leaf expansion and fruit set and development, such as observed in Dre and other deciduous fruit trees, possibly due to a deficit in C assimilation. The subsequent C reserve recovery in these trees is suggested to occur when peak leaf production coincides with declined C demand by sink tissues [54].

Nevertheless, the contribution of leaf Suc and starch as supplies for the strong sink demands imposed by flowering and fruiting in *Diopyros* trees remain to be determined, considering that the mechanisms governing C mobilization and re-allocation from carbohydrate storage reserves to carbohydrate utilizing sink tissues in trees are poorly understood, particularly in deciduous trees [52]. Another aspect that needs to be examined is why NSC contents were so different in leaves of perennial and deciduous *Diopyros* trees. The latter, considering that a recent analysis of the seasonal variation of foliar NSCs in leaves and other tissues, that compared deciduous broad-leafed, deciduous conifer and evergreen conifer trees, found that foliar NSC concentrations and seasonal NSC fluctuations, particularly from June to October, were surprisingly similar between them [57].

However, data regarding foliar sucrolytic enzyme activity in *Diopyros* trees (Fig 5) provided an insight on the possible role played by these enzymes in their C mobilization processes. For instance, the gradual reduction in Suc leaf content in Ddg that commenced in the summer, could be attributed, at least partially, to the peak in CWI activity observed in this season (Fig 5A and 5C), and to the transient activation of VI and CI activities occurring in the spring of 2015 (not shown). In addition, and as mentioned above, Suc depletion might have been an indication of its export to sink tissues to support the C mobilization needed to sustain the demands of the flowering and fruit growth processes that occurred from the autumn onwards. This contrasted with the invertase activity pattern observed in Dre, in which the transient peak accumulation of Suc and starch accumulation in the summer coincided with a significantly diminished leaf CWI (Fig 5B and 5D) and, perhaps, CI activities (not shown). Contrary to Ddg, no VI was detected in leaves of Dre. Also of interest was the absence of SuSy activity detected in leaves of these trees, regardless of species or season. The physiological implications of this conspicuous absence remain to be defined, considering the proposed role of SuSy enzymes in, for example, stress amelioration responses, phloem loading and sucrose utilization in the laticifers, in poplar and/ or rubber trees [58]. A possible explanation for their absence in leaves of *Digyna* trees may be related to the alleged inhibition of SuSy activity by invertases that has been reported to occur in other trees as a consequence of limiting sucrose availability [59]. Additionally, studies performed in *Robinia pseudoacacia* and poplar trees have revealed that SuSy activity is mostly detected in strong sinks such as developing and differentiating woody tissues [60, 61], or in developing citrus or apple fruits [62, 63]. On the other hand, the differences in CWI activity observed between both species, which were clearly lower in leaves on Ddg, irrespective of the season, might have been an indication of maturity in perennial leaves, similarly to observations made in oak, citrus and poplar, where the activity of acid invertases declined with increasing leaf age [64].

Invertase activity might have been also associated with the release of hexoses required for the biosynthesis of secondary metabolites and, perhaps, for the protection of the trees against abiotic stress. Thus, the accumulation of hexoses observed in the winter 2014-spring 2015, possibly as a residual result of the high CWI activity detected in the winter, could explain the build-up of secondary metabolites, mostly flavones (Fig 7), observed in leaves of Dre. As mentioned above, seasonal foliar CWI activity in these trees was approximately 2- to 5-fold higher than that detected in leaves of Ddg, where secondary metabolite accumulation was much lower. This disparity, coupled with supporting information derived from a study in leaves of *Labisia pumila*, a Malaysian medicinal herb, where a significant correlation was found between K^+^-enhanced acid and alkaline invertase activity and total phenolics and flavonoid content [65], is in favor of the proposed relationship between secondary metabolite accumulation and invertase activity in Dre. Thus, similarly to *L*. *pumila*, the increased conversion of sucrose to hexoses by high invertase activity in Dre might have increased glucose availability for the enhanced production of these metabolites. Likewise, several additional studies have found that phenylalanine ammonia-lyase, a key enzyme involved in the phenylpropanoid pathway involved in the biosynthesis of flavonoids, coumarins and many other metabolites is regulated by the variation of the sucrose/ hexose ratio [66]. In contrast, the clearly lower level of these secondary metabolites in leaves of Ddg in seasons of the year when hexoses were abundant suggest that these might have been employed for other purposes, perhaps as osmolytes to protect them against the atypical low temperatures registered in the autumn-winter in Taretan (Fig 1). In this regard, the higher accumulation of leaf soluble NSCs and starch contents together with increased sucrolytic and sucrose synthesizing enzyme activities detected in two contrasting perennial *Sabina* tree species was also interpreted as a protective response against freezing stress [67]. Another interesting aspect is that the accumulation of phenolic acids, known to have antioxidant activity and photo-protective properties [68] also coincided with the high PPFDs received by the leaves of Dre trees in the spring of 2015 (S4 Table). Therefore, the light-intensity-related accumulation of secondary metabolites observed in leaves of Dre was in agreement with several studies that have reported that changes in light intensity may influence the accumulation of secondary metabolites in several aromatic and medicinal plant species. These include, apart from phenolics and flavonoids, volatiles and alkaloids [34, 36, 69, 70].

## Conclusions

This field study validated the proposed working hypothesis by revealing that several leaf biochemical parameters such as total chlorophyll, Chl a/ Chl b ratio, NSCs, protein and secondary metabolite content varied in a species-specific and season-dependent manner in two contrasting *Diospyros* trees. Some of these differences, such as NSC content fluctuations associated with C mobilization, could be partly attributed to their perennial/ evergreen or deciduous growing habit. Meanwhile, others could be ascribed as part of the tree’s protective responses against excess illumination and/ or other abiotic stresses such as lower than normal temperatures. In addition, the variation in the intra-specific responses among individual trees indicated that leaf biochemistry in leaves of *Diopyros* can be markedly plastic as a means to adapt to variations in their growing environment. Another important aspect of this study was the variability between the responses produced by trees of the same species, irrespective of the season examined. We suggest that two prevalent factors could have contributed to the lack of uniform responses observed between individual tree specimens: i) the orientation of the trees selected for study, which in addition to the different vegetation composition present in the two locations examined, caused them to receive different light intensities, and ii) the lack of uniformity in the soil characteristics of both locations. In this respect, the highly contrasting soil composition observed in the Teocuitatlán de Corona site, where Dre trees were sampled, was most probably due to the fact that they grew separately in a ravine with a 20 m height differential, which could have promoted micro-climatic conditions and/ or specific ecological niches for each specimen.

## Supporting information

S1 Fig Correlation matrix of biochemical and environmental variables in leaves of *Diospyros digyna* trees sampled during the spring (**A**), summer (**B**), autumn (**C**) and winter (**D**) of 2015.
(PDF)

S2 Fig Correlation matrix of biochemical and environmental variables in leaves of *Diospyros rekoi* trees sampled during the spring (**A**), summer (**B**), autumn (**C**) and winter (**D**) of 2015.
(PDF)

S3 Fig Principal component analysis showing which environmental factors (shown as vectors of varying size) most significantly influenced the overall biochemical variables (shown as dots) of *Diospyros digyna* leaves. (PDF)

S4 Fig Principal component analysis showing which environmental factors (shown as vectors of varying size) most significantly influenced the overall biochemical variables (shown as dots) of *Diospyros rekoi* leaves. (PDF)

S1 Table Mean seasonal irradiation and long-wave radiation recorded in Taretan, Michoacán, México, *D*. *digyna’s* sampling site. (PDF)

S2 Table Mean seasonal irradiation and long-wave radiation recorded in Teocuitatlán de Corona, Jalisco, México, *D*. *reloi’s* sampling site. (PDF)

S3 Table Seasonal variation in photosynthetic photon flux density (PPFD) recorded in five (T1-to-T5) *Diospyros digyna* (Ddg) trees sampled in Taretan, Michoacán, México. (PDF)

S4 Table Seasonal variation in photosynthetic photon flux density (PPFD) recorded in five (T1-to-T5) *Diospyros rekoi* (Dre) trees sampled in Teocuitatlán de Corona, Jalisco, México. (PDF)

S5 Table Correlations between photosynthetic photon flux density (PPFD) and Chla/ Chlb ratios determined in five (T1-to-T5) *Diospyros digyna* (Ddg) trees for the winter 2014-15-winter 2015–16 period. Numbers in red indicate statistically significant correlations.
(PDF)

S6 Table Correlations between photosynthetic photon flux density (PPFD) and Chla/ Chlb ratios determined in five (T1-to-T5) *Diospyros rekoi* (Dre) trees for the winter 2014-15-winter 2015–16 period. Numbers in red indicate statistically significant correlations.
(PDF)

## Abbreviations

Ddg: *Diospyros digyna*
Dre: *Diospyros rekoi*
PPFD: photosynthetic photon flow density
C_T_: total chlorophyll content
Chla/ Chlb: chlorophyll a/ chlorophyll b ratio
