# Supplementary material for: Seasonal variation in non-structural carbohydrates, sucrolytic activity and secondary metabolites in deciduous and perennial Diospyros species sampled in Western Mexico
Source: PLoS One. 2017 Oct 26;12(10):e0187235. doi: 10.1371/journal.pone.0187235 (PMC5658181; doi:10.1371/journal.pone.0187235)
Supplement: S2 Fig — Correlation matrix of biochemical and environmental variables in leaves of Diospyros rekoi trees sampled during the spring (A), summer (B), autumn (C) and winter (D) of 2015. (PDF) [file pone.0187235.s002.pdf]

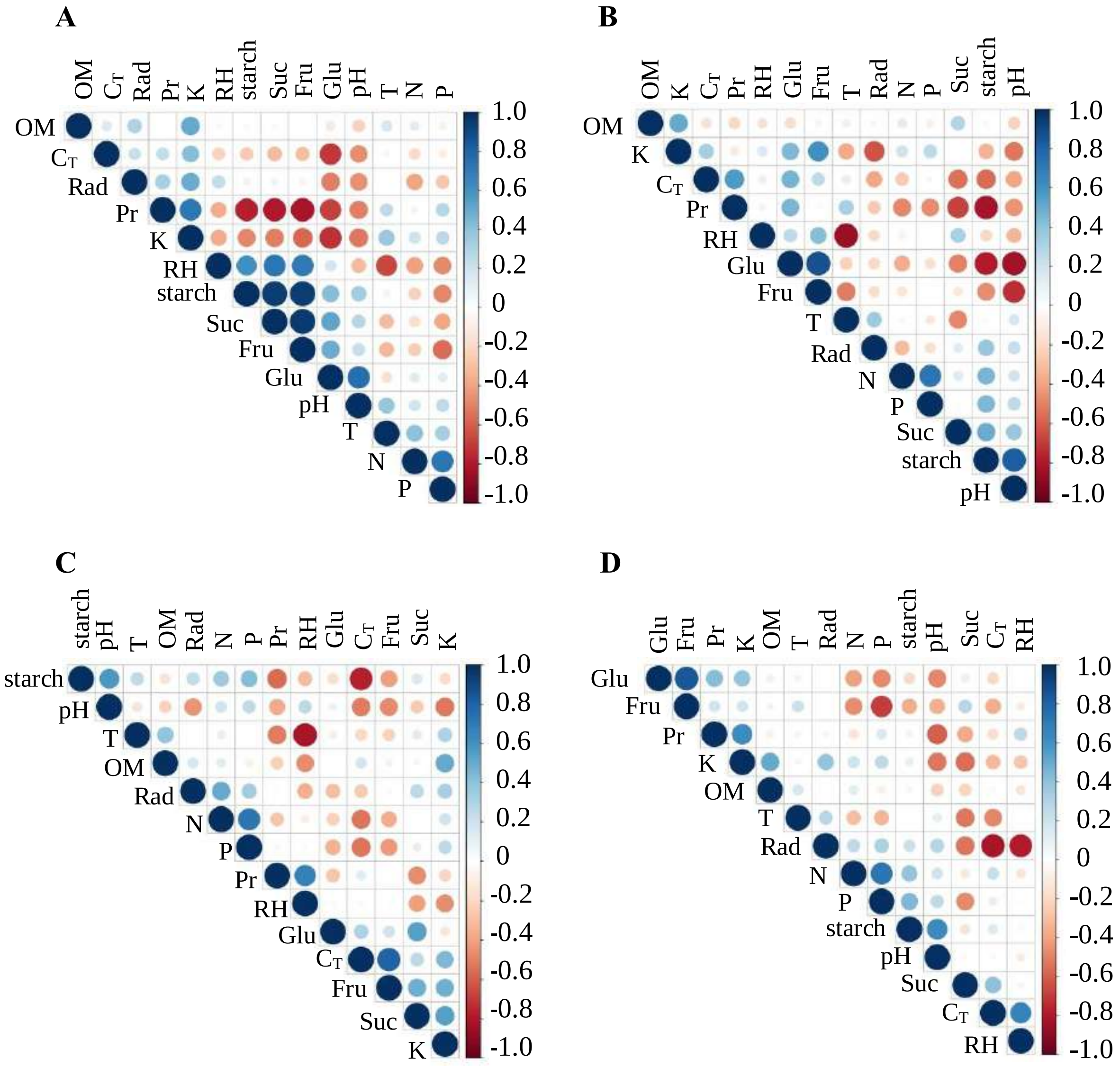

Figure S2. Correlation matrix of biochemical and environmental variables in leaves of *Diospyros rekoi* trees sampled during the spring (**A**), summer (**B**), autumn (**C**) and winter (**D**) of 2015. The biochemical variables represented are the following: starch, Suc (sucrose), Glu (glucose), Fru (fructose), Pr (protein), and total chlophyll (C_T). The environmental variables are: N (soil nitrogen), P (soil phosphorus), K (soil potassium), OM (soil organic matter), pH (soil pH), T (temperature), RH (relative humidity), and Rad (solar radiation). Positive and negative correlations are shown in blue and red, respectively, whereas the size and color intensity of the dots are directly proportional to their statistical significance.
