# Supplementary material for: Seasonal variation in non-structural carbohydrates, sucrolytic activity and secondary metabolites in deciduous and perennial Diospyros species sampled in Western Mexico
Source: PLoS One. 2017 Oct 26;12(10):e0187235. doi: 10.1371/journal.pone.0187235 (PMC5658181; doi:10.1371/journal.pone.0187235)
Supplement: S4 Fig — (PDF) [file pone.0187235.s004.pdf]

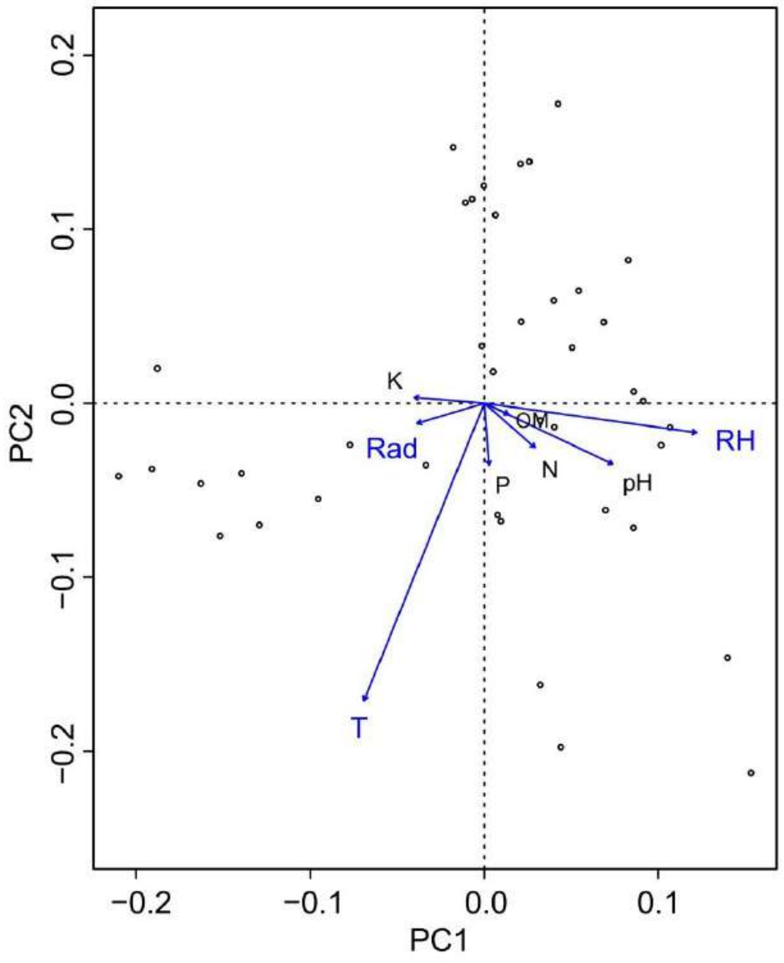


**Figure S4.** Principal component analysis showing which environmental factors (shown as vectors of varying size) most significantly influenced the overall biochemical variables (shown as dots) of *Diospyros rekoi* leaves. Influential environmental variables, shown in decreasing order of magnitude, are: T (temperature); RH (relative humidity); pH (soil pH); Rad (solar radiation); N (soil nitrogen content); P (phosphorus soil content); K (potassium soil content), and OM (soil organic matter).
