# Supplementary material for: Seasonal variation in non-structural carbohydrates, sucrolytic activity and secondary metabolites in deciduous and perennial Diospyros species sampled in Western Mexico
Source: PLoS One. 2017 Oct 26;12(10):e0187235. doi: 10.1371/journal.pone.0187235 (PMC5658181; doi:10.1371/journal.pone.0187235)
Supplement: S1 Table — (PDF) [file pone.0187235.s005.pdf]

Table S1. Mean seasonal irradiation and long-wave radiation recorded in Taretan, Michoacán, México, *D. digyna's* sampling site.

		MJ/ m^2 /day ($\dot{X}$ seasonal)	
		Irradiation (horizontal Surface)	Long Wave
**2014-15**	Winter	15.458	29.258
	Spring	37.259	20.074
**2015**	Summer	38.645	14.719
	Autumn	31.948	16.429
**2015-16**	Winter	27.946	15.775
