# Supplementary material for: Seasonal variation in non-structural carbohydrates, sucrolytic activity and secondary metabolites in deciduous and perennial Diospyros species sampled in Western Mexico
Source: PLoS One. 2017 Oct 26;12(10):e0187235. doi: 10.1371/journal.pone.0187235 (PMC5658181; doi:10.1371/journal.pone.0187235)
Supplement: S2 Table — (PDF) [file pone.0187235.s006.pdf]

Table S2. Mean seasonal irradiation and long-wave radiation recorded in Teocuitatlán de Corona, Jalisco, México, *D. reloi's* sampling site.

		**MJ/ m²/ day (Ẋ seasonal)**	
		Irradiation (horizontal Surface)	Long Wave
**2014-15**	Winter	13.496	28.290
	Spring	37.438	19.438
**2015**	Summer	38.790	15.313
	Autumn	31.507	15.317
**2015-16**	Winter	27.541	13.494
