# Supplementary material for: Seasonal variation in non-structural carbohydrates, sucrolytic activity and secondary metabolites in deciduous and perennial Diospyros species sampled in Western Mexico
Source: PLoS One. 2017 Oct 26;12(10):e0187235. doi: 10.1371/journal.pone.0187235 (PMC5658181; doi:10.1371/journal.pone.0187235)
Supplement: S3 Table — (PDF) [file pone.0187235.s007.pdf]

Table S3. Seasonal variation in photosynthetic photon flux density (PPFD) recorded in five (T1-to-T5) *Diospyros digyna* (Ddg) trees sampled in Taretan, Michoacán, México.

		PPFD (Ddg)				
		**T1**	**T2**	**T3**	**T4**	**T5**
**2014-15**	Winter	765	542	723	90.4	77.5
		763	544	722.5	91	75
		765	541.5	720	89.9	76.3
	Spring	163.5	1431.7	1033	77.5	70
		166	1432	1040	76	66
		162.7	1430	1036	77.2	65
**2015**	Summer	1398.5	1410.3	1494	88	105
		1400	1410.5	1492	86.8	100
		1402	1415	1493.8	87.5	103.8
	Autumn	1378	1696	406	69	42
		1376.5	1697	404.7	72	40
		1380	1695	405	71	41.4
**2015-16**	Winter	343.7	62.6	60	115	60.5
		347	64	57.6	116.3	60.57
		348.1	66	58.8	116.8	59.8
