# Supplementary material for: Seasonal variation in non-structural carbohydrates, sucrolytic activity and secondary metabolites in deciduous and perennial Diospyros species sampled in Western Mexico
Source: PLoS One. 2017 Oct 26;12(10):e0187235. doi: 10.1371/journal.pone.0187235 (PMC5658181; doi:10.1371/journal.pone.0187235)
Supplement: S4 Table — (PDF) [file pone.0187235.s008.pdf]

Table S4. Seasonal variation in photosynthetic photon flux density (PPFD) recorded in five (T1-to-T5) *Diospyros rekoi* (Dre) trees sampled in Teocuitatlán de Corona, Jalisco, México.

		PPFD (Dre)				
		**T1**	**T2**	**T3**	**T4**	**T5**
**2014-15**	Winter	1297	4256	1671	955	733
		1299	4256.4	1668	956	738
		1287.6	4256.9	1670	955.7	730
	Spring	1360	2309	1960.5	1121	1779
		1368.5	2311	1962	1123	1783
		1365.3	2309	1959.6	1126	1787
**2015**	Summer	1170.5	35	127	106.7	106.39
		1172	32.9	127.5	105.2	106.4
		1165	33.7	126.8	104	107.3
	Autumn	54	72	157.5	37.3	32.5
		57	71	157.9	38	31.1
		60	69	156	37.9	29.7
**2015-16**	Winter	1540	4910	1880	1016.4	1476
		1539	4905	1879	1018	1470.8
		1542	4906.5	1878.7	1014	1475
