# Supplementary material for: Seasonal variation in non-structural carbohydrates, sucrolytic activity and secondary metabolites in deciduous and perennial Diospyros species sampled in Western Mexico
Source: PLoS One. 2017 Oct 26;12(10):e0187235. doi: 10.1371/journal.pone.0187235 (PMC5658181; doi:10.1371/journal.pone.0187235)
Supplement: S5 Table — Numbers in red indicate statistically significant correlations. (PDF) [file pone.0187235.s009.pdf]

Table S5. Correlations between photosynthetic photon flux density (PPFD) and Chla/ Chlb ratios determined in five (T1-to-T5) *Diospyros digyna* (Ddg) trees for the winter 2014-15-winter 2015-16 period. Numbers in red indicate statistically significant correlations.

		Chla/ Chlb (Ddg)				
	**PPFD**	**T1**	**T2**	**T3**	**T4**	**T5**
**2014-15**	Winter	0.755	0.765	-0.795	-0.707	0.737
	Spring	-0.711	-0.623	-0.910	0.804	-0.785
**2015**	Summer	-0.424	-0.274	-0.06	-0.362	-0.389
	Autumn	0.036	0.426	0.402	-0.487	-0.268
**2015-16**	Winter	-0.173	-0.189	0.809	-0.817	0.926
