# Supplementary material for: Seasonal variation in non-structural carbohydrates, sucrolytic activity and secondary metabolites in deciduous and perennial Diospyros species sampled in Western Mexico
Source: PLoS One. 2017 Oct 26;12(10):e0187235. doi: 10.1371/journal.pone.0187235 (PMC5658181; doi:10.1371/journal.pone.0187235)
Supplement: S6 Table — Numbers in red indicate statistically significant correlations. (PDF) [file pone.0187235.s010.pdf]

Table S6. Correlations between photosynthetic photon flux density (PPFD) and Chla/ Chlb ratios determined in five (T1-to-T5) *Diospyros rekoi* (Dre) trees for the winter 2014-15-winter 2015-16 period. Numbers in red indicate statistically significant correlations.

		Chla/ Chlb (Dre)				
	**PPFD**	**T1**	**T2**	**T3**	**T4**	**T5**
**2014-15**	Winter	0.052	0.121	0.019	0.028	0.058
	Spring	-0.053	0.053	-0.111	0.077	-0.121
**2015**	Summer	-0.120	-0.131	-0.019	-0.251	-0.861
	Autumn	0.604	-0.673	-0.028	-0.157	-0.957
**2015-16**	Winter	0.013	0.581	-0.971	0.371	-0.181
